# Metabolomics Biomarkers: A Strategy Toward Therapeutics Improvement in ALS

**DOI:** 10.3389/fneur.2018.01126

**Published:** 2018-12-18

**Authors:** Débora Lanznaster, Denis Reis de Assis, Philippe Corcia, Pierre-François Pradat, Hélène Blasco

**Affiliations:** ^1^Université de Tours, Inserm U1253, Tours, France; ^2^Centre Constitutif SLA, CHRU Bretonneau, Tours, France; ^3^Federation des centres SLA de Tours et Limoges, LITORALS, Tours, France; ^4^Département des Maladies du Système Nerveux, Centre Référent Maladie Rare SLA, Hôpital de la Pitié-Salpétrière, Paris, France; ^5^Laboratoire d'Imagerie Biomédicale, Sorbonne Université, CNRS, INSERM, Paris, France; ^6^Northern Ireland Centre for Stratified Medicine, Biomedical Sciences Research Institute Ulster University, C-TRIC, Altnagelvin Hospital, Londonderry, United Kingdom; ^7^Service de Biochimie et Biologie Moléculaire, CHRU de Tours, Tours, France

**Keywords:** ALS, metabolomics, pharmacometabolomics, therapeutic, creatinine

## Abstract

Biomarkers research in amyotrophic lateral sclerosis (ALS) holds the promise of improving ALS diagnosis, follow-up of patients, and clinical trials outcomes. Metabolomics have a big impact on biomarkers identification. In this mini-review, we provide the main findings of metabolomics studies in ALS and discuss the most relevant therapeutics attempts that targeted some prominent alterations found in ALS, like glutamate excitotoxicity, oxidative stress, alterations in energetic metabolism, and creatinine levels. Metabolomics studies have reported putative diagnosis or prognosis biomarkers, but discrepancies among these studies did not allow validation of metabolic biomarkers for clinical use in ALS. In this context, we wonder whether metabolomics knowledge could improve ALS therapeutics. As metabolomics identify specific metabolic pathways modified by disease progression and/or treatment, we support that adjuvant or combined treatment should be used to rescue these pathways, creating a new perspective for ALS treatment. Some ongoing clinical trials are already trying to target these pathways. As clinical trials in ALS have been disappointing and considering the heterogeneity of the disease presentation, we support the application of a pharmacometabolomic approach to evaluate the individual response to drug treatments and their side effects, enabling the development of personalized treatments for ALS. We suggest that the best strategy to apply metabolomics for ALS therapeutics progress is to establish a metabolic signature for ALS patients in order to improve the knowledge of patient metabotypes, to choose the most adequate pharmacological treatment, and to follow the drug response and side effects, based on metabolomics biomarkers.

## Introduction

Amyotrophic lateral sclerosis (ALS) is the most common adult-onset motor neuron disease, which ultimately leads to death due to respiratory failure usually 3–5 years after the appearance of first symptoms. ALS wandering diagnosis spreads ~12 months after symptoms onset—this long delay being partly related to the lack of specific diagnostic tests. Today, only two pharmacological treatments are approved for ALS: riluzole and edaravone, which only show small effects on survival and decline of functional impairment, respectively. Numerous clinical trials have been conducted on the identification of new therapies for ALS, but their findings are disappointing. One of the reasons of these failures could be the use of inappropriate methodology in the clinical studies, like poor design or lack of appropriate cohort enrichment strategies (1). Early diagnosis could also increase recruitment of patients in earlier stages of the disease to clinical trials (2). Moreover, the functional scales used to assess motor function in ALS patients (ALS Functional Rating Scale-Revised; ALSFRS-R, forced vital capacity, and muscular testing) may be insensitive to subtly follow drug response. Thus, the search and identification of reliable biomarkers for ALS diagnosis and prognosis is of utmost importance, as biomarkers follow-up could help in the identification of drug-response phenotypes, improving evaluation of treatment efficacy.

“Omics” research comprise systemic analyses (including transcriptomics, genomics, proteomics, lipidomics, and metabolomics) that advanced immensely in the field of biomarkers. For example, proteomics research identified a structural neuronal protein, the neurofilament, as a putative biomarker for ALS, especially for ALS diagnosis regarding its sensitivity and specificity (3). Neurofilaments also showed promising results in the field of prognostic prediction factors (4–6), but its application was not yet validated in the clinical practice.

Metabolomics studies identified several metabolites related to pathways implicated in the pathophysiology of ALS, both in animal models and in ALS patients, thus improving our knowledge about the disease mechanisms (7, 8). These metabolites could represent ALS biomarkers alone or in combination, by composing a metabolic signature for ALS. Furthermore, as identified metabolites are related to pathways that are modified in the disease, adjuvant therapy could target these pathways, and compensate their dysfunction. Identification of metabolic signatures also enables a personalized therapy and the direct follow up of drug effect in each patient—a proposition of a new field called pharmacometabolomics. In this review, we provide the main findings of metabolomics studies in ALS for biomarkers identification or for understanding ALS pathophysiology. Furthermore, we summarize recent evidence that support metabolomics applications in the clinical practice, as improvement of therapeutics and treatment follow-up. Here, we shed a light into other applications of metabolomics knowledge through the extension of its interest beyond the biomarkers research.

## What Can Metabolomics Analyses Tell Us?

Metabolomics is based on the global search for metabolites, defined as small molecules that represent the downstream products of ongoing biological processes in cells, tissues, and other biological samples (9). A particular metabolic profile—or “metabotype”—of a systemic biofluid (such as blood or the cerebrospinal fluid, CSF) reflects directly the metabolic status of different organs and tissues because of continuous exchanges of metabolites between tissues and fluids (7). To design a metabolic profile, metabolites are selected according to their polarity, mass, and concentrations using high-throughput techniques (10). After data pre-treatment, metabolites are analyzed by univariate analysis and multivariate analysis to identify the most important contributors to the discrimination between samples (11, 12).

Metabolomics research identified several individual metabolites and metabolic signatures (with or without identification of each metabolite composing such signature) that can discriminate ALS from non-ALS cases (10, 13–16). Metabolomics can also determine metabolic signatures that identify distinct subgroups of ALS patients according to their clinical characteristics or disease evolution (17–19). Altogether, the main objectives of metabolomics studies performed in ALS have been punctually reached. However, its application in the clinical routine or its extension to other aims (for example, for following drug responses) will depend on the ability to overcome several limitations of the method—for example, the differences in samples treatment, data analysis, and lack of external validation for many of these identified signatures.

## Metabolomics Studies Identified Metabolites Related With Pathophysiological Mechanisms in ALS

Although the exact mechanism that initiate ALS pathogenesis remain partially unknown, glutamatergic excitotoxicity, oxidative stress, and mitochondrial dysfunction have been reported as key contributors to the motor neuron degeneration (20). Metabolomics may provide a new light to evaluate these pathophysiological pathways by identifying metabolites directly associated with these pathways (8). Here we summarize the main findings of metabolomics studies linked with the most prominent pathophysiological alterations observed in ALS patients. Interestingly, these alterations were also observed in ALS models.

### Glutamate

Glutamate plays a key role in ALS, as it is not only involved in excitotoxicity, but also in other mechanisms such as oxidative stress and metabolism disturbance (21). The only treatment approved that counteract the glutamatergic hyperactivation in ALS is riluzole, a non-competitive blocker of glutamatergic transmission (22–24). Glutamate remains the most cited metabolite increased in blood samples (12, 25, 26) and CSF (25, 27–29) from ALS patients, as reported by independent research groups. Recently, a metabolomics study proposed glutamic acid as a potential biomarker for ALS, after validating it in a healthy cohort (30). The increase of glutamate in CSF could be linked with the decrease in astrocytic glutamate transporter (GLT)-1 expression in motor cortex and spinal cord observed in ALS patients (17, 31, 32). Interestingly, ALS animal models also present alterations in glutamate levels (33–35). Rats expressing the ALS-linked familial mutation Super Oxide Dismutase-1 (SOD1)-G93A showed a decrease in the astrocytic glutamate transporter expression in the spinal cord (36), as reported in ALS patients. Is important to note that astrocytes have been pointed as key elements in the pathophysiology of ALS, as is for their role in mediating glutamatergic activation or as for their metabolic support to neurons (37).

### Antioxidants

Oxidative stress is also a well-known mechanism involved in ALS and is directly linked with glutamatergic toxicity that increases the production of reactive oxygen species (ROS) (38, 39). Astrocytes release ascorbic acid (an endogenous antioxidant) after glutamatergic stimulation, and the elevated level of ascorbate in the CSF of ALS patients may reflect a compensatory mechanism (11, 40). Another antioxidant metabolite, uric acid, was shown to be involved in ALS pathophysiology. Increased levels of uric acid were suggested to be associated with a slow progression of ALS (41, 42). Homocysteine, another endogenous antioxidant, was also pointed by metabolomics studies as a potential biomarker for ALS (30, 39).

### Lipids

ALS patients usually present compromised energy homeostasis, including basal hypermetabolism, body weight loss, and abnormal metabolism of glucose and lipids (43, 44). In agreement with that, ALS patients present a 10-fold increase in the cholesterol esters C16:0 and C18:0 in the spinal cord, while in a mice model of ALS these substances are increased by 4- and 10-fold in the lower spinal cord during the presymptomatic and symptomatic phases, respectively (45). *Postmortem* analyses show that the spinal cord tissue from ALS patients presents a remarkable decrease in docosahexaenoic acid (DHA) levels and in n-3 polyunsaturated fatty acids (PUFA), in sharp contrast with the increase of DHA content found in the brain cortex (46).

### Creatinine

Reduced levels of creatinine in the CSF or blood from ALS patients were reported from different research groups, including metabolomics studies (42, 47–49). Creatinine reflects skeletal muscle production and reduced levels of this metabolite are directly related to amyotrophy, a cardinal ALS symptom. Use of plasma creatinine levels as a biomarker in ALS was suggested for monitoring disease progression in clinical trials (50), and creatinine was the first metabolite already used to evaluate drug therapy response to dexpramipexole in a clinical trial (51).

Findings regarding metabolomics are promising but disappointing, as, to date, no biomarker was approved for diagnosis or prognosis use (10). To go further with this approach, well-designed and large cohorts studies would be essential for biomarker validation (52), and the improvement of analytical and statistical steps may improve the robustness of the strategy (16, 19). Importantly, all metabolomics studies published so far have identified metabolites linked to the same pathophysiological pathways, thus reinforcing the potential of metabolomics to explain pathophysiological mechanisms underlying ALS. In this context, we suggest that metabolomics analyses may be useful for other applications than identifying diagnostic or prognostic biomarkers, such as for example, monitoring disease course and identifying treatment outcomes and side effects in clinical trials.

## Metabolomics-Identified Alterations as Targets For New Therapeutic Strategies

Disturbed pathways identified through metabolomics studies in cellular and animal models, as well as in ALS patients, hold the potential to be used for the discovery of new therapies in ALS (48). The application of metabolomics findings in preclinical and clinical studies to target glutamatergic toxicity (21) and energy metabolism dysfunction (44) were already reviewed. Thus, here we will summarize the ongoing therapeutics attempts that target alterations identified by metabolomics studies and with beneficial effects in ALS preclinical tests (Table 1).

**Table 1:** Ongoing clinical trials with therapeutics interventions focused in alterations identified by metabolomics studies.

**Target**	**Intervention**	**Clinical trials for ALS**
Glutamatergic overactivation	Perampanel	Phase II, NCT03377309 (Lebanon); NCT03019419 (Japan); NCT03020797 (Unites States).
	Memantine	Ongoing (phase II, NCT02118727, Unites States). [No effect observed in phase II-III; (53)].
Oxidative stress	Inosine	Phase I, NCT02288091 (United States).
	CC100	Phase I, NCT03049046 (United States).
Hypermetabolism	Triheptanoin	Phase I-II, NCT03506425 (United States).
	High caloric fatty diet	NCT02306590 (Germany).
	Oral nutritional supplementation (high fat and protein)	NCT02152449 (France).

*Information available in clinicaltrials.gov*.

As mentioned before, metabolomics and non-metabolomics studies demonstrated alterations in glutamate levels in CFS and blood of ALS patients. Several clinical trials tried to demonstrate the effect of anti-glutamatergic drugs—already approved for the treatment of other neurological diseases—for the treatment of ALS, but failed to show any improvements. This is the case for lamotrigine, topiramate, gabapentin, and talampanel (21). Current active clinical trials investigate the potential effect of memantine and perampanel in ALS, drugs used for Alzheimer's disease and epilepsy treatment, respectively (21).

Focusing on oxidative stress (as edaravone, the recent drug approved by the FDA for ALS treatment that is a ROS scavenger), a clinical trial is investigating the effect of inosine treatment for ALS. Inosine is a precursor of uric acid, an antioxidant molecule that is found altered in ALS patients. Furthermore, this clinical trial will follow therapy response by analyzing uric acid levels in treated individuals, applying metabolomics approaches both at treatment strategy and follow-up. CC100 (a synthetic form of the caffeic acid phenethyl ester) is another molecule with antioxidant properties that is currently being investigated in a Phase I clinical trial. The caffeic acid phenethyl ester is a natural compound with effects on lipid peroxidation and lipid metabolism (54).

Considering that energy metabolism is also altered in ALS patients, several studies focused in providing additional fuel to increase energy uptake (44). While preclinical studies successfully showed the beneficial effects of these treatments, clinical trials failed to show the same results. In the case of dexpramipexole (an improver of oxidative phosphorylation and thus of ATP synthesis), a Phase II clinical trial showed prevention of functional decline of ALS patients following a 12-month treatment (55). However, Phase III failed to show improvements (56). A Phase II clinical trial performed between 2009 and 2012 analyzed the beneficial effects of two hypercaloric (one high-fat and other high-carbohydrate) diets in ALS patients receiving enteral nutrition. Patients receiving a high-carbohydrate enteral formula presented less adverse effects compared to control subjects. They found that both diets were safe and tolerable, although they did not modify disease progression (57). Currently, ongoing clinical trials investigate the effect of high caloric fatty supplementation (Calogen®) and high caloric protein/fat supplementation (Fortimel®) in ALS patients.

Novel therapeutic strategies may focus on creatinine as a marker to identify the efficacy of drugs and follow-up of treatments aiming the inhibition of the muscular loss observed in ALS, or even in treatments aiming the increase of muscle mass in the patients. For example, in ALS animal models, inhibition of myostatin (a negative regulator of muscle growth) improved muscular mass and strength. Although myostatin treatment did not change the disease onset and progression, it improved the muscular function, especially in the diaphragm of the animals (58). If translated for the human disease, it could improve the quality of life of ALS patients during disease progression.

## Metabolomics-Driven Therapeutics Management: The Advent of Pharmacometabolomics

Metabotype information can be used to identify alterations in biochemical pathways in ALS patients that are modified or not by treatment. This new field, called pharmacometabolomics, allows clinicians to identify a metabolic state at baseline and after drug therapy, increasing information about treatment outcomes, especially drug-response phenotype (59).

Different studies revealed the potential of pharmacometabolomics to assess drug therapy response and identify distinct signatures of metabolites before and after treatment exposure in diverse pathologies, from cancer to cardiovascular diseases. For ALS, one study analyzed metabolites and lipids composition of plasma samples from individuals enrolled in a phase III clinical trial for Olexosime. This study identified a metabolic profile that distinguished the placebo from the Olexosime group, characterized mainly by alterations in the levels of glycine, citrulline/arginine, and kynurenine. Furthermore, clinical progression of ALS correlated with amino acids, lipids, and spermidine levels in the Olexosime group, and with glutamine levels in the placebo group (19). It is noteworthy to highlight that these metabolites are linked with some of the pathological pathways involved in ALS pathology (glutamatergic alteration and energy metabolism dysfunction), as described before.

In practice, pharmacometabolomics findings may improve the strategy of drug administration scheme, as a complementary tool of pharmacokinetics, and may provide new light on drug-response effect and downstream signaling pathways (60). This information may provide details on biochemical pathways involved in disease and in treatment effect in ALS patients in a narrowly controlled process.

## Metabolomics Research in ALS Should Improve Therapeutics—Concluding Remarks

Metabolomics represent a new approach that is increasingly gaining importance as it helps to identify biomarkers and unravels pathways that contribute to the pathophysiology of ALS. Significant therapeutic advances are based on a deep knowledge of ALS pathogenesis and metabolomics holds great potential to play a key role in this objective. However, despite the efforts made by metabolomics researchers to identify biomarkers for ALS, no biomarker was validated yet. Metabolomics studies should rather focus in identifying metabolic signatures then individual biomarkers for ALS. This would be a revolutionary step toward developing efficient strategies to evaluate not only disease progression, but also treatment responses to drug therapies (19).

This also point out the urgent need of metabolomics research to combine analysis and information (1) of different tissues in ALS patients, as CSF, blood and muscle samples; and (2) by combining different approaches (proteomics, transcriptomics, lipidomics, etc.) (52). Combination of “omics” approaches with clinical evaluation (for example, ALSFRS-R) could be the best practice for an early diagnosis of ALS (10). Importantly, omics analysis should be standardized between different research centers together with refinement of statistical analysis tools to analyze better the results obtained. Altogether, these efforts should readily improve metabolomics application in the daily clinical practice.

Metabolomics can also be applied to identify outcomes of pharmacological treatment. Usual parameters and endpoints used in clinical trials to evaluate drug efficacy are probably not enough sensitive to observe a slight effect. In this regard, metabolomics could identify biomarkers that are sensitive enough to detect even small effects of drugs tested in Phase II clinical trials, allowing them to be investigated into Phase III. Furthermore, pharmacometabolomics approaches provide help in evaluating drug effect as a primary or additional parameter. Metabolome may provide longitudinal, reproducible, and objective data that are crucial criteria to evaluate drug effect. Besides, adjuvant therapy based on metabolomics findings could compensate the identified altered pathways in a subtype of patients, allowing a personalized therapeutic strategy targeting specifically these pathways. Ongoing trials using this strategy are presented in Table 1. However, no study yet tried to approach several pathways at once, using a combined therapeutic strategy. This approach should be more relevant than focusing only on one altered pathway.

**Figure 1:**
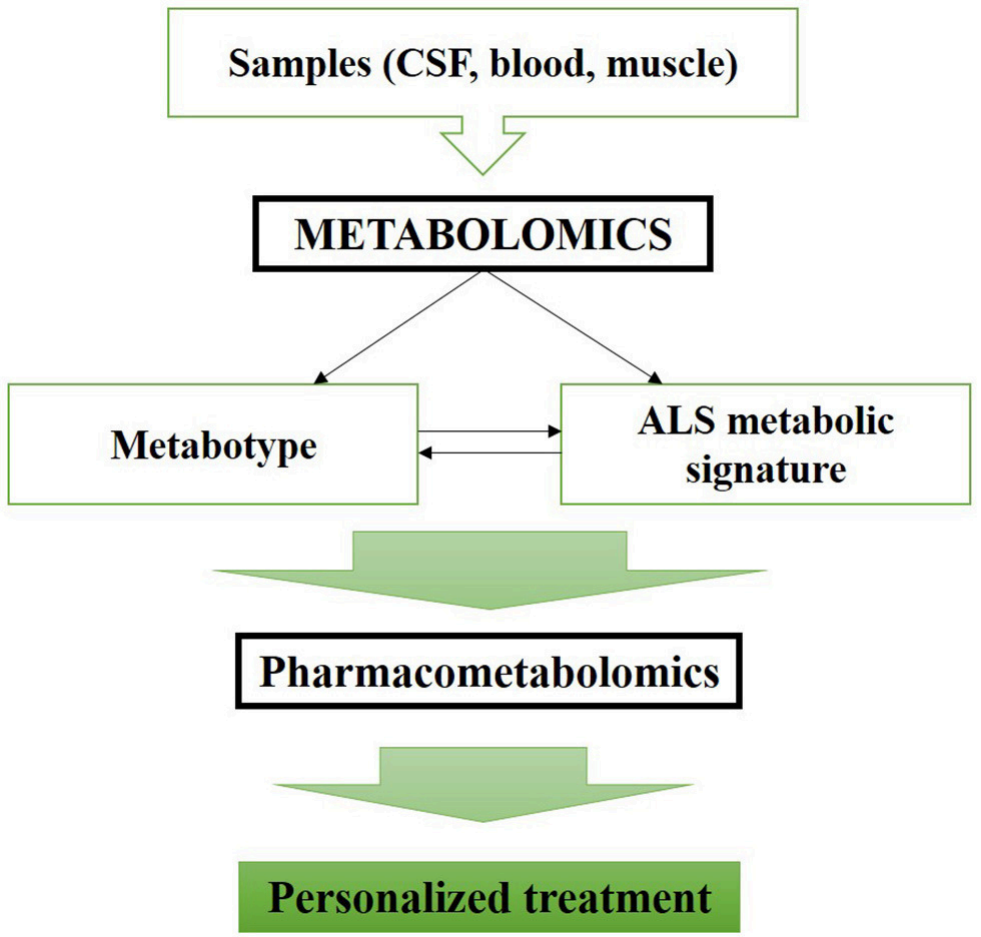
Metabolomics applicability enhance ALS therapeutic management and allows a personalized medicine.

Metabolomics applied early in ALS management should improve therapeutic strategy and development. The major interest of metabolomics at disease onset is to build homogeneous subgroups of patients in order to apply a personalized therapeutic approach (Figure 1). Metabolomics complement data obtained from genomics, transcriptomics and proteomics, and combined with pharmacometabolomics approaches, they add the final piece of information to the study of disease pathophysiology and drug response (60). We propose to combine omics and clinical data to improve our comprehension about the specific metabolic pathways affected in each individual patient. Stratification of patients based on all these findings would considerably improve trials methodology and care management, as well as therapeutics strategies by providing a mean to a personalized medicine. To our knowledge, this review is the first to present diagnosis and prognosis biomarkers as an initial step to develop therapeutics. This new light on metabolomics application is promising for complex and heterogeneous diseases, like ALS, characterized by successive therapeutics failures.

## Author Contributions

DL and HB wrote the manuscript. DL, DRA, PC, PFP and HB critically revised the manuscript for important intellectual content. All authors read and approved the submitted version.

### Conflict of Interest Statement

The authors declare that the research was conducted in the absence of any commercial or financial relationships that could be construed as a potential conflict of interest.
